# Ocular counter-roll is less affected in experienced versus novice space crew after long-duration spaceflight

**DOI:** 10.1038/s41526-022-00208-5

**Published:** 2022-07-20

**Authors:** Catho Schoenmaekers, Chloë De Laet, Ludmila Kornilova, Dmitrii Glukhikh, Steven Moore, Hamish MacDougall, Ivan Naumov, Erik Fransen, Leander Wille, Steven Jillings, Floris L. Wuyts

**Affiliations:** 1 Lab for Equilibrium Investigations and Aerospace, University of Antwerp, Antwerpen, Belgium; 2 Institute of Biomedical Problems, Moscow, Russia; 3 Central Queensland University, School of Engineering and Technology, St Lucia, QLD Australia; 4 University of Sydney School of Psychology, Sydney, NSW Australia; 5 StatUa Center for Statistics, University of Antwerp, Antwerpen, Belgium

**Keywords:** Neuroscience, Physiology, Neurology

## Abstract

Otoliths are the primary gravity sensors of the vestibular system and are responsible for the ocular counter-roll (OCR). This compensatory eye torsion ensures gaze stabilization and is sensitive to a head roll with respect to gravity and the Gravito-Inertial Acceleration vector during, e.g., centrifugation. To measure the effect of prolonged spaceflight on the otoliths, we quantified the OCR induced by off-axis centrifugation in a group of 27 cosmonauts in an upright position before and after their 6-month space mission to the International Space Station. We observed a significant decrease in OCR early postflight, larger for first-time compared to experienced flyers. We also found a significantly larger torsion for the inner eye, the eye closest to the rotation axis. Our results suggest that experienced cosmonauts have acquired the ability to adapt faster after G-transitions. These data provide a scientific basis for sending experienced cosmonauts on challenging missions that include multiple g-level transitions.

## The human vestibular organ: a multisensory system

Humans highly depend on the vestibular organs, located bilaterally in the inner ear, as they detect head movements and transmit this information to the brain to ensure upright posture, gaze stabilization, and spatial orientation^1^. The vestibular organs consist of the semicircular canals (SCCs) that are stimulated by angular accelerations, and the otoliths that detect the sum of linear accelerations acting upon the head. This vector sum is referred to as the Gravito-Inertial Acceleration (GIA) vector.

**Fig. 1:**
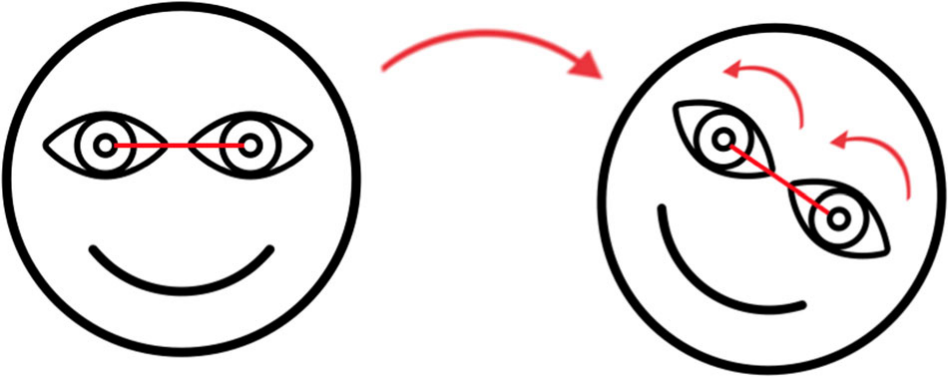
Visual representation of the ocular counter-roll (OCR). The OCR tends to rotate the eyes in the opposite direction of the head roll. For example, when a head roll to the right is performed, the eyes will rotate to the left as a compensatory reflex.

The otoliths are the primary graviceptors of the vestibular organ by registering linear accelerations on the one hand, including gravitational acceleration, and lateral tilts of the head on the other hand. Otoliths transmit their information to the brain to determine the spatial vertical, which is essential for controlling our eye movements and posture. An important otolith-mediated ocular reflex is the ocular counter-roll (OCR) that is generated when the head is laterally tilted, e.g., while driving around a corner or during centrifugation^2–4^. The OCR tends to rotate the eyes in the opposite direction to the roll tilt and toward the GIA^5^ (Fig. 1). Although this reflex is imperfect, since the angle of the rotated eye is smaller than that of the induced tilt, it allows us to maintain gaze stabilization and postural stability, e.g., when making sharp turns during locomotion.

To get a better understanding of the importance of otolith-mediated reflexes, we study them in relation to spaceflight. Cosmonauts in the International Space Station (ISS), orbiting around Earth, are subjected to microgravity or <10^−6^ g. The lack of gravitational input will influence the otolith’s function by decreasing the gain (ratio of eye torsion over head tilt) of otolith-mediated reflexes, indicating a deconditioning of the otoliths^6^. Also, the assessment of the real vertical will be impaired when there is a loss of otolith input to the brain in microgravity^7^. Deconditioning of otolith-mediated reflexes following microgravity exposure has been proposed as one of the multiple causing factors of the postural, locomotor, navigational, and gaze control problems as well as orthostatic intolerance (OI) experienced by returning astronauts^8^. OI is the inability to regulate blood pressure, usually in a standing position, which can induce a (pre)syncope or fainting. These symptoms are generally maintained as long as the otoliths are not yet readapted to Earth’s gravitational level of 1 g^6^. The OCR is therefore an important measure to understand neurovestibular adaptation due to spaceflight since it isolates the effect of otolith adaptations.

The OCR reflex has been used in several studies as a measure of the effect of microgravity on the otoliths^8–14^. It should be noted that there are conflicting results from studies showing a decrease, increase or no change in OCR upon return compared to the preflight OCR^15^. However, these studies often had a small sample size, due to general difficulties and limitations of access to space crew, and due to difficulties in recording torsional eye movements. Moreover, the results were mostly from short-duration spaceflights, making it difficult to generalize these results to prolonged exposure to microgravity. For example, Clément et al. studied seven cosmonauts who went to space for 10–13 days. On Earth, the cosmonauts were rotated along their longitudinal axis and were tilted 10° and 20° off-axis, generating an OCR. However, OCR measurements on the day of landing and up to 4 days postflight did not significantly differ from preflight measurements^15^. Another study, by Kornilova et al., studied a group of 13 cosmonauts with long-duration missions (126–196 days), of whom they obtained OCR measurements by having the cosmonauts tilt their heads sideways 30–35°. They observed a decrease in OCR as measured on the first 2 days after return compared to preflight, which recovered to baseline 8–9 days after landing^16^. Lastly, our group has also demonstrated a decrease in OCR by off-axis centrifugation 3 days after landing, which returned to baseline values as measured 9 days postflight. That study included 25 OCR measurements at each pre- and postflight timepoint, with cosmonauts spending on average 186 days in space^6^.

The current study extends our previous research on OCR changes after long-duration spaceflight, of which results have previously been published^6^. The first aim of this study is to report the OCR changes induced by 6-month spaceflights in an extended dataset compared to our previous work. Note that we have now analyzed nearly double the amount of data compared to our previous study and that for the current study, all data were analyzed by the same person to exclude operator bias. The second aim is to determine whether previous experience in space influences the postflight OCR response, which has not yet been investigated. In total, 27 cosmonauts (13 first-time flyers, 14 experienced flyers) took part in this study, several of whom participated in multiple ISS missions (11 cosmonauts participated twice, 1 participated three times, and 1 participated four times). This resulted in a total of 44 longitudinal datasets, where a longitudinal dataset comprises two preflight measurements as a baseline data collection (BDC1 and BDC2), and up to three postflight measurements grouped as 1–3 days (*R* + 1/3), 4–7 days (*R* + 4/7), and 8–12 days (*R* + 8/12) after spaceflight. These 44 longitudinal datasets contained data of 13 first-time, 16 second-time, 8 third-time, 4 fourth-time, and 3 fifth-time flyers. The OCR is induced using an off-axis centrifuge with the cosmonauts seated upright, facing the direction of rotation (right-ear-out during counterclockwise (CCW) and left-ear-out during clockwise (CW) rotation), and with a fixed distance of 0.5 m from the rotation axis. The cosmonauts were first centrifuged for 5 min in a CCW and subsequently in a CW direction. The OCR was recorded during constant angular velocity to purely obtain measurements of otolith function.

## Methods

### Experiment timeline and subjects

The test used to induce the OCR was the Visual and Vestibular Investigation System (VVIS) located in the Gagarin Cosmonaut Training Centre in Star City near Moscow, Russia. We investigated 27 cosmonauts (*N* = 13 first-time flyers, *N* = 14 experienced flyers), several of whom were tested twice or even more times during consecutive spaceflight missions to the ISS (*N* = 15 were tested once, *N* = 11 were tested twice, *N* = 1 was tested three times, *N* = 1 was tested four times). As a result, 44 cosmonaut experiments were performed, 31 were conducted for frequent flyers, while the other 13 experiments were conducted for the remaining cosmonauts who were first-time flyers (*N* = 13 first-time flyers, *N* = 16 second-time flyers, *N* = 8 third-time flyers, *N* = 4 fourth-time flyers, and *N* = 3 fifth-time flyers).

The cosmonauts were tested before and after their 6-month space mission in the ISS between ISS increment mission 16 in October 2007 to increment 61 in April 2020. The preflight experiments consisted of 2 baseline recordings defined as the BDC (BDC1 *N* = 44, and BDC2 *N* = 40), and the postflight experiments consisted of 2–3 recordings of the OCR. The first postflight measurement was taken 1–3 days after the return to Earth, defined as *R* + 1/3 (*N* = 32). The second postflight measurement, which was not recorded for all cosmonauts, was taken 4–7 days after their return to Earth, defined as *R* + 4/7 (*N* = 23). The third postflight measurement was taken 8–12 days after their return to Earth and is therefore defined as *R* + 8/12 (*N* = 39). It was impossible to test all the cosmonauts on the same day after their return, due to medical and organizational limitations.

The experiment protocol was designed in accordance with the ethical standards defined in the 1964 Declaration of Helsinki and was accepted by Human Research Multilateral Review Board (HRMRB) and European Space Agency (ESA). Cosmonauts gave their written informed consent prior to their voluntary participation in this study.

### Visual and Vestibular Investigation System (VVIS)

**Fig. 2:**
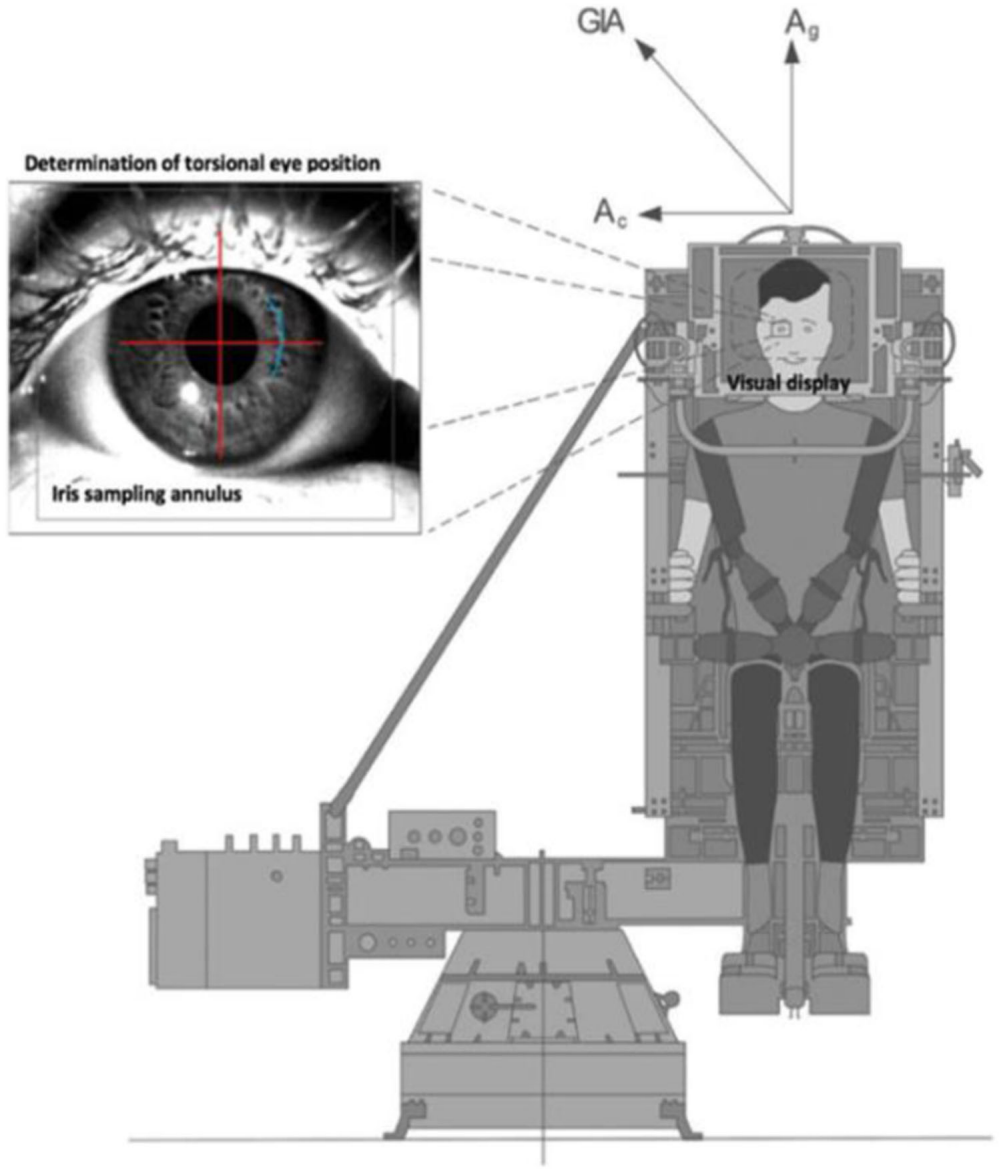
Representation of experimental setup. The net linear acceleration stimulating the otoliths, during off-axis centrifugation, is the vector sum of the gravitational (Ag) and centripetal acceleration (Ac), termed the Gravito-Inertial Acceleration (GIA). When GIA was interpreted as the spatial vertical during centrifugation, the cosmonaut should experience a sensation of 45° tilt (Reprinted from ref. ^8^ with permission of Macmillan Publishers Ltd, copyright 2015).

The cosmonauts were seated upright on the rotation chair, 0.5 m away from the vertical rotation axis, and securely fastened by a five-point safety harness with a restriction of head movements (Fig. 2). The experimentation room was darkened to avoid any visual motion feedback or fixation during rotation. A visual display, mounted in front of the cosmonaut’s face, was used to project visual targets during parts of the experiment. Binocular three-dimensional video-oculography with infrared video goggles was used to enable continuous recordings of dynamic changes in ocular torsion. 3D video-oculography is a non-invasive method for recording the horizontal, vertical, and torsional components of eye movements.

**Fig. 3:**
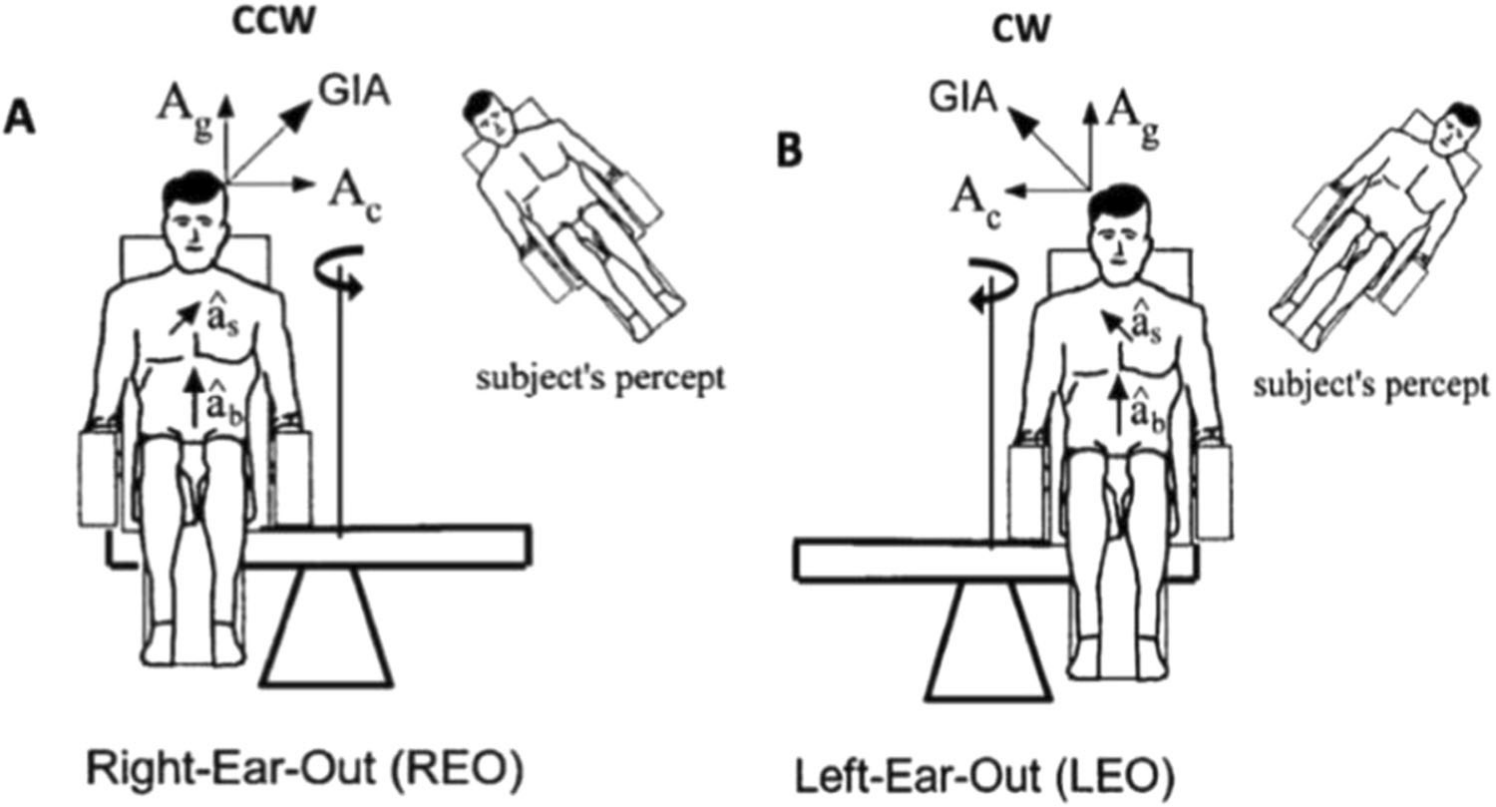
The rotation axis is placed next to the subject, meaning that both otoliths will be simultaneously stimulated by GIA. **A** When the subject was moving according to the counterclockwise (CCW) direction, a virtual tilt of 45° was experienced to the right (right-ear-out, REO). **B** When the subject was moving according to the clockwise (CW) direction, a virtual tilt of 45° was experienced to the left (left-ear-out, LEO)^6,46^ (From Moore, 2001^46^ with permission; reproduced from Experimental Brain Research).

At standstill, the calibration of the video goggles and a baseline recording were performed. After an acceleration phase of 30°/s^2^, the cosmonaut was subjected to a constant angular velocity of 254°/s resulting in an outward centripetal acceleration Ac of 1 g first for 5 min in a CCW direction. The chair was decelerated with a rate of 3°/s^2^ to standstill. The chair was then manually 180° rotated, and subsequently, the protocol was repeated for 5 min in a CW direction. In between both centrifugation directions, the cosmonaut remains seated while the operator changes the centrifuge configuration to the subsequent (CW) direction. The cosmonaut faced the direction of motion, with the right ear outwards during CCW rotation and the left ear outwards for the CW rotation. The vector sum of the gravitational acceleration Ag and the centripetal acceleration Ac is called the GIA. This GIA was perceived by the subject as the ‘spatial vertical’ and exerted a shear force on the otolith system which caused an illusory or virtually perceived roll tilt of 45° during rotation. As a result, an OCR was induced that tended to orient the eyes toward the GIA and thus away from the direction of the perceived tilt. The virtual tilt was outwards, meaning that the cosmonaut had the impression of tilting to the right when moving CCW and to the left when moving CW. This means that the eyes will rotate toward the left during CCW centrifugation and to the right during the CW centrifugation, always toward the axis of rotation (Fig. 3).

### OCR measurements

**Fig. 4:**
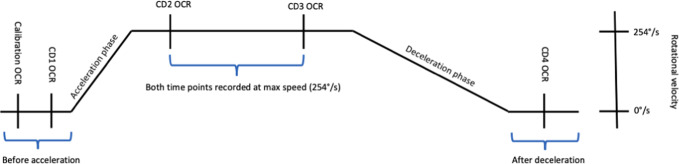
Visual overview of conducted measurements. Before acceleration, at a standstill, the calibration and the first OCR measurement (CD1) were performed. After an acceleration phase of 30°/s, when the maximal rotational velocity of 254°/s was reached during rotation, the second and third measurements of the OCR (CD2 and CD3) were performed. After the cessation of a 3°/s deceleration phase, at standstill, the last OCR measurement was performed (CD4).

The OCR measurements were taken before, during, and after centrifugation according to a fixed protocol. The first and fourth OCR measurements were respectively taken before and after centrifugation during standstill, where no centripetal force was acting upon the body and thus an OCR of 0° was observed as expected. The second OCR measurement was taken 40 s after the steady-state phase of constant rotational velocity was reached, because we only wanted to assess the contribution of the otolith system to the OCR. During the 40 s, the cupula of the horizontal SCCs returns to its original position and no longer contributes to the OCR. The third OCR measurement was taken 40 s before the start of the deceleration phase. The time interval between the second and third OCR measurements was 3 min and 40 s, during which other protocols were applied during centrifugation. During centrifugation, the second and third OCR measurement, an OCR of on average 5–7°^17^ was expected to be measured because of GIA stimulating the otoliths. Each OCR measurement was recorded for 20 s, while the cosmonaut observed a fixation or central dot (CD) on the visual display. The CD was used to cancel out other eye movements, e.g., saccades and nystagmi, during centrifugation. It is important to mention that the CD could prevent horizontal eye motion that may be otolith generated and should therefore be included as a limitation. The OCR was calculated as the difference of the average eye torsion over these 20 s recordings, consisting of 600–1000 frames, between rotation and standstill (Fig. 4).

The video files obtained during the VVIS experiment contain recordings of the eye movements and were analyzed in a visual programming language (custom made by H.M. in LabVIEW – National Instruments −11500 N Mopac Expwy. Austin, TX, USA) to measure the OCR in degrees. The OCR was calculated for both eyes and centrifugation directions (CCW and CW). As part of quality control, we selectively removed artifactual data arising from eye blinks and eyelashes.

### Statistical analysis

The OCR measurements were statistically analyzed in JMP^®^ (version Pro 16. SAS Institute Inc, Cary, NC, 1989–2001), with *p* < 0.05 as the significance threshold. All sample sizes for our models can be found in Table 1. We built three linear mixed models (LMM) using a stepwise forward approach^18,19^. We first tested our main variables as fixed effects and then systematically tested all interaction terms. The variables included were Timepoint (BDC1, BDC2, *R* + 1/3, *R* + 4/7, and *R* + 8/12), Days After Return (range 1–12), Flight (a cosmonaut’s amount of space missions, range 1–5), Eye (left and right), and Orientation (CCW and CW). The significance threshold used for selecting the fixed effect was set at *p* = 0.001. Non-significant terms were removed until all combinations were tested and only the significant ones remained. In all models, Cosmonaut was entered as a random intercept to account for the non-independence between observations from the same cosmonaut. Random slope terms Cosmonaut*Flight and Cosmonaut*Flight*Timepoint were added as random effects in case these terms significantly improved the fit of the model, as tested using the Likelihood ratio test. The residuals of all models were checked for normality and homoskedasticity.

**Table 1:** Overview OCR values.

		CCW left, inner, eye			CCW right, outer, eye			CW left, outer, eye			CW right, inner, eye		
		Mean	SEM	Valid *N*	Mean	SEM	Valid *N*	Mean	SEM	Valid *N*	Mean	SEM	Valid *N*
OCR	BDC1	6.15°	0.08	44	5.53°	0.07	44	5.53°	0.10	44	5.89°	0.11	44
	BDC2	6.02°	0.10	40	5.45°	0.08	40	5.41°	0.10	38	5.94°	0.11	38
	*R* + 1/3	3.45°	0.17	32	3.05°	0.17	33	2.86°	0.15	32	3.29°	0.17	32
	*R* + 4/7	4.37°	0.10	23	3.88°	0.10	23	3.63°	0.10	23	4.08°	0.14	23
	*R* + 8/12	6.03°	0.08	39	5.38°	0.09	40	5.42°	0.08	38	5.88°	0.11	39

This table gives an overview of all OCR values at CD2 for each timepoint (BDC1, BDC2 (two preflight measurements), *R* + 1/3, *R* + 4/7, and *R* + 8/12 (three postflight measurements)), centrifugation direction (CCW and CW), and eye (left and right eye).

Our first model evaluated the factors influencing the OCR measurements pre- and postflight, to investigate the effect of long-duration spaceflight. The categorical variable Timepoint was used as a fixed effect, defining two preflight measurements (BDC1, BDC2) and three postflight measurements (*R* + 1/3, *R* + 4/7, *R* + 8/12). The random slope term (Cosmonaut*Flight*Timepoint) and the random intercept terms (Cosmonaut and Cosmonaut*Flight) were kept in the model according to their Likelihood ratio tests (*p* < 0.0001 for all). Given the significant term Eye*Orientation, we created a new interaction term Eye_Rotation defining the inner and outer eye with respect to the rotational axis of the centrifuge. Dunnett’s correction for multiple testing was used for the post hoc analysis to compare OCR at the first baseline measurement (BDC1) to each of the following timepoints.

Our second model evaluated how the OCR measurements were evolving in the first week after return from a space mission (selection threshold: Days after return <8), the readaptation to the normal gravitational level of Earth. The random slope term (Cosmonaut*Flight*Timepoint) and the random intercept terms (Cosmonaut and Cosmonaut*Flight) were kept in the model according to their Likelihood ratio tests (*p* < 0.0001 for all). The continuous variable Days After Return was entered as a fixed effect to model the effect of the days since return from a space mission.

To investigate the effect of the previous spaceflight, or experience, on the otolith-mediated OCR^20^, we modeled the impact of the number of previous spaceflights (variable Flight) on the changes observed in OCR at *R* + 1/3. For this analysis, we entered the percent change in OCR at *R* + 1/3 compared to BDC1 as a dependent variable in the model. The number of flights was entered as a fixed effect using a piecewise linear model with a breakpoint at Flight = 2. The position of the breakpoint was based on visual inspection and our previous results. The regression lines were fitted using a LMM. the random intercept terms (Cosmonaut and Cosmonaut*Flight) were kept in the model according to their Likelihood ratio tests. The model for the mean is:

1 $${{{\mathrm{Y}}}} = \beta _{{{\mathrm{0}}}} + \beta _{{{\mathrm{1}}}} \ast {\rm{x}} + \beta _{{{\mathrm{2}}}} \ast \left( {{{{\mathrm{x}}}} - {{{\mathrm{k}}}}} \right)^{{{\mathrm{ + }}}}$$

Equation 1, where *Y* is the percentage change in OCR at *R* + 1/3 and *k* = 2.

*X* is the number of flights the cosmonaut has experienced.

The regression line is made of two segments with a changing slope beyond two flights. The first segment (for Flight ≤ 2) has the equation: *y* = *β*_0_ + *β*_1_*x, with *β*_1_ estimating the change in *Y* per extra flight.

The second segment (for Flight > 2) has equation: *y* = *β*_0_ + *β*_1_*x + *β*_2_*(*x* – *k*). In this equation, *β*_2_ estimates the change in slope beyond Flight = 2. The significance of *β*_2_ tests whether this change is significant.

The piecewise linear analysis generates two regression slopes, the first one applicable for the flight variable (from 1 to 5) and the second one for data of cosmonauts with more than two previous spaceflights. We then test if that second slope is different from zero. If not, the second slope does not bring any additional information to the regression and the evolution of the data is the same before and after the inflection point.

### Reporting summary

Further information on research design is available in the Nature Research Reporting Summary linked to this article.

## Results

Table 1 presents the absolute values of the OCR measurement for each timepoint, centrifugation direction (CCW and CW), and eye (left and right eye), whereas the Supplementary figure contains the raw OCR data trace of one cosmonaut at preflight (BDC1) and 3 days after return (*R* + 3).

Since OCR values were recorded on different occasions within the same cosmonauts, the modeling of OCR changes was carried out in an LMM framework. All sample sizes can be found in Table 2 and all estimates can be found in Table 3. All given results are provided with standard errors.

**Table 2:** Overview of the valid number of data points for each flight, rotation, eye, and timepoint.

	CCW												
	Right						Left						All CCW
	BDC1	BDC2	*R* + 1/3	*R* + 4/7	*R* + 8/12	All right CCW	BDC1	BDC2	*R* + 1/3	*R* + 4/7	*R* + 8/12	All left CCW	
F1	13	12	9	10	13	57	13	12	8	10	12	55	112
F2	15	14	12	5	12	58	15	13	12	5	12	57	115
F3	8	6	5	4	7	30	8	7	5	4	7	31	61
F4	4	4	3	3	4	18	4	4	3	3	4	18	36
F5	3	3	3	1	3	13	3	3	3	1	3	13	26
All	43	39	32	23	39	176	43	39	31	23	38	174	350

	CW												
	Right						Left						All CW
	BDC1	BDC2	*R* + 1/3	*R* + 4/7	*R* + 8/12	All right CW	BDC1	BDC2	*R* + 1/3	*R* + 4/7	*R* + 8/12	All left CW	
F1	13	12	8	10	13	56	13	12	8	10	12	55	111
F2	15	12	12	5	12	56	15	12	12	5	12	56	112
F3	8	6	5	4	6	29	8	6	5	4	6	29	58
F4	4	4	3	3	4	18	4	4	3	3	4	18	36
F5	3	3	3	1	3	13	3	3	3	1	3	13	26
All	43	37	31	23	38	172	43	37	31	23	37	171	343

This table represents the timepoints as the intervals, for each separate day the valid sample size is: *R* + 1 *N* = 4, *R* + 2 *N* = 9, *R* + 3 *N* = 116, *R* + 4 *N* = 16, *R* + 5 *N* = 68, *R* + 6 *N* = 8, *R* + 7 *N* = 0, *R* + 8 *N* = 12, *R* + 9 *N* = 70, *R* + 10 *N* = 44, *R* + 11 *N* = 18, *R* + 12 *N* = 12.

*Fx* flight number x*, CW* clockwise*, CCW* counterclockwise*, BDC* baseline data collection*, R* *+* *X* return after *X* days*.*

**Table 3:** Overview of the specifications or estimates of the different linear mixed models (LMM).

Fixed effects	Estimate (°)	Std error (°)	CI 95% (°)
*Model 1: the effect of long-duration spaceflight on the otolith-mediated reflex, the OCR*			
Intercept	5.78	0.12	[5.55; 6.02]
Timepoint [BDC2]	0.07	0.16	[−0.24; 0.40]
Timepoint [*R* + 1/3]	−3.76	0.18	[−4.12; −3.40]
Timepoint [*R* + 4/7]	−1.96	0.18	[−2.31; −1.60]
Timepoint [*R* + 8/12]	0.09	0.16	[−0.22; 0.41]
Orientation [CW]	−0.15	0.04	[−0.23; −0.07]
Flight	0.14	0.06	[0.01; 0.26]
Eye_Rotation [Outer Eye]	−0.49	0.04	[−0.57; −0.41]
Timepoint [BDC2]*Flight	−0.07	0.08	[−0.21; 0.11]
Timepoint [*R* + 1/3]*Flight	0.50	0.09	[0.32; 0.67]
Timepoint [*R* + 4/7]*Flight	0.12	0.09	[−0.07; 0.31]
Timepoint [*R* + 8/12]*Flight	−0.07	0.08	[−0.23; 0.09]
*Model 2: the OCR re-adapts to the normal gravitational level of Earth*			
Intercept	−0.26	0.38	[−1.01; 0.48]
Flight	1.03	0.15	[0.75; 1.32]
Orientation [CW]	−0.23	0.07	[−0.38; −0.09]
Eye_Rotation [Outer Eye]	−0.41	0.07	[−0.56; −0.27]
DaysAfterReturn	0.80	0.09	[0.63; 0.97]
DaysAfterReturn*Flight	−0.14	0.03	[−0.21; −0.07]
*Model 3: the effect of previous spaceflight on the otolith-mediated OCR changes*			
Intercept	0.18	0.05	[0.09; 0.28]
Flight	0.19	0.03	[0.14; 0.25]
Flight >2	−0.16	0.04	[−0.24; −0.08]

BDC1 was taken as the reference value.

*BDC* baseline data collection, *R* + *X* return after *X* days.

### The effect of long-duration spaceflight on the otolith-mediated reflex, the OCR

**Fig. 5:**
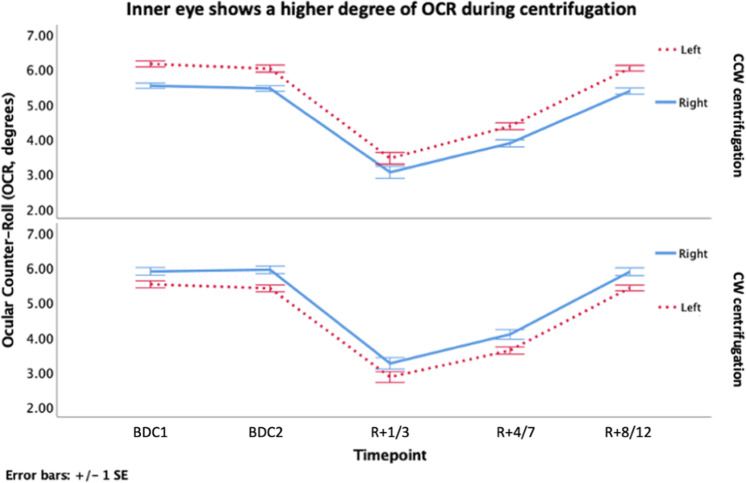
The inner eye shows a higher degree of OCR during centrifugation. During counterclockwise (CCW) centrifugation, the left eye is defined as the inner since it is the closest to the axis of rotation. This graph shows that the left, inner, eye shows a higher degree of torsion during CCW centrifugation. During clockwise (CW) centrifugation, the right, inner, eye shows a higher degree of OCR compared to the left, outer, eye. Baseline data collection (BDC, preflight), return after X days (*R* + X, postflight), CCW, and CW. Error bars represent standard error of mean with multiplier one.

**Fig. 6:**
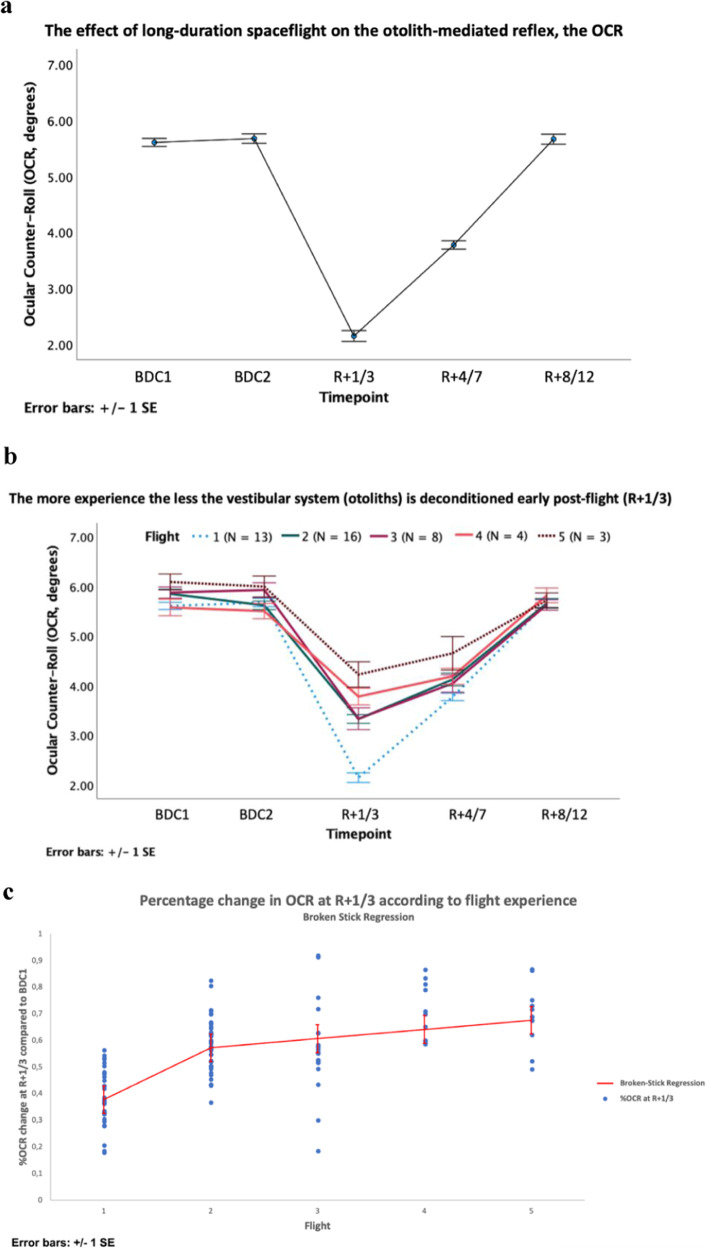
The effect of long-duration spaceflight and spaceflight experience on the otolith-mediated reflex, the OCR. **a** The effect of spaceflight on the otolith-mediated reflex, the ocular counter-roll (OCR). The overall average (including both eyes and centrifugation directions) of the OCR measurements at CD2 for the first-time flyers show a decreased OCR early postflight (*R* + 1/3) after a 6-month space mission. In addition, the evolution of the OCR across the first 2 weeks postflight is illustrated, showing a return to BDC levels 8–12 days after their return. **b** The difference between 1st-, 2nd-, 3rd-, 4th-, and 5th-time flyers regarding the effect of long-duration spaceflight on the otolith-mediated OCR. All flyers (first-time to frequent flyers) show a clear decrease in early postflight (*R* + 1/3). However, the more the cosmonauts have flown the less the OCR gain is decreased postflight. For each flier, the OCR measurement at CD2 is represented as an average of both centrifugation directions and eyes. **c** Percentage change in OCR at *R* + 1/3 according to the flight experience. Piecewise linear regression. BDC baseline data collection, *R* + X return after X days. Error bars of the three graphs represent standard error of mean with multiplier one.

Our first model evaluated the OCR measured for both eyes across different timepoints (BDC1, BDC2, *R* + 1/3, *R* + 4/7, and *R* + 8/12), different rotation directions (CCW and CW), and different experience levels of the cosmonauts (first- to fifth-time flyers). The effect of the inner vs outer eye with respect to the rotational axis of the centrifuge proved to be significant with the eye closest to the axis of rotation (the inner eye) showing a significantly higher OCR (0.49 ± 0.04°) compared to the eye furthest away from the axis of rotation (the outer eye) (Fig. 5). The centrifugation direction also influences the OCR in this model. Finally, we showed an effect of previous spaceflight experience on the OCR, more in particular on the OCR changes between timepoints (Fig. 6). The model showed an effect of the timepoints on the OCR. All fixed effects had a significance level of *p* < 0.001 (LMM), and the residuals were normally distributed. The cosmonaut’s age when in space appeared as a non-significant effect in this model.

**Fig. 7:**
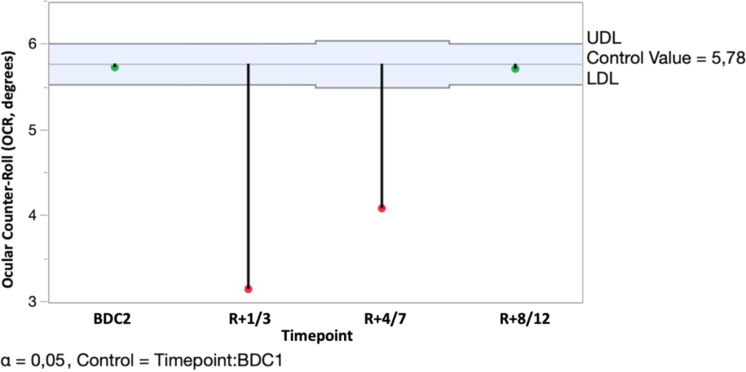
The post hoc Dunnett’s test. Timepoint BDC1 was taken as the reference, this post hoc analysis shows the significant difference between the first two postflight (*R* + 1/3 and *R* + 4/7), whereas both preflight (BDC2) and last postflight (*R* + 8/12) measurements are not significantly different.

In addition, a post hoc analysis with Dunnett’s correction was performed to compare OCR between BDC1 and all subsequent measurements. The second baseline measurement of the OCR (BDC2) was not different from BDC1, proving a good test-retest reliability of the data. The OCR significantly decreased early postflight at *R* + 1/3 and *R* + 4/7 compared to baseline (BDC1). At *R* + 8/12, OCR was back to preflight levels (BDC1) (Fig. 7). This shows a clear decline in OCR early postflight that tends to normalize in the first week after return.

Finally, the precision of the method to measure the OCR was tested using Levene’s test of Equality of Error variances on the cohort of first-time flyers (to avoid group inhomogeneity) revealing no difference in variances (*p* = 0.23) (Levene’s test of Equality of Error variances) at the five different timepoints. Hence, spaceflight had no effect on the precision of the OCR data collected.

### The OCR re-adapts to the normal gravitational level of Earth

Our second model then evaluated the readaptation process of the OCR occurring early after the return. As it appeared that the OCR was back to baseline levels at *R* + 8/12, this next analysis focused on the first 7 days after return from a space mission using the days after return as a continuous independent variable. As we expected, this model showed similar results to the first one with comparable estimates for flight experience, rotation direction, and inner/outer eye as significant fixed effects. In addition, the model highlighted a readaptation process occurring early postflight with an increase in OCR of 0.80 ± 0.09° per day after return. Interestingly, the model suggested different recovery slopes across cosmonauts according to their experience levels. Per flight achieved by a cosmonaut, the OCR recovery per day after landing is less steep by a factor of 0.14 ± 0.03°. All fixed effects shown had a significance level of *p* < 0.001 (LMM), and the residuals were normally distributed.

### The effect of previous spaceflight missions on the otolith-mediated OCR changes

The results from the previous model tend to suggest a difference in the OCR decrease at *R* + 1/3 depending on flight experience, with a larger decrease for first-time flyers compared to more experienced flyers. Moreover, the difference in postflight OCR changes between first and second flight data seemed higher than between data from an increasing number of space missions (third, fourth, and fifth flight data). With the current model, we evaluated the impact of previous flight experience on the OCR changes at *R* + 1/3, while also looking at a possible different trend after the second flight. Therefore, we fitted a piecewise linear regression model on the percentage change in OCR at *R* + 1/3 compared to BDC1. Given our previous results, we hypothesized a different change in OCR after the second flight and used two previous flights as the breaking point.

The piecewise linear analysis generates two regression slopes, the first one applicable for the original flight variable (from 1 to 5) and the second one for data of cosmonauts with more than two previous spaceflights (Flight > 2). We then test if that second slope is different from zero. If not, the second slope does not bring any additional information to the regression and the evolution of the data is the same before and after the breaking point.

The first slope shows the change in the dependent variable (percent drop in OCR at *R* + 1/3 compared to baseline) per extra flight. The second slope coefficient models the change in slope beyond two flights, and tests whether this change is significantly different from zero. If not, the slope beyond Flight 2 is not significantly different from the slope before Flight 2.

In this model, similarly to the results of the previous model, the first slope showed a smaller change in OCR at *R* + 1/3 of 0.19 ± 0.03% less per flight previously flown. More experienced flyers have a less decreased OCR at *R* + 1/3. Moreover, the second slope being significant in our model (*p* value = 0.0003) (piecewise linear analysis) proved a different trend in change across flight experience. After the second flight, the change in OCR at *R* + 1/3 was reduced by 0.16 ± 0.04% leaving only 0.3% change in OCR per flight after the second flight. The largest change in OCR early postflight is seen between the first and the second spaceflight. The changes in OCR early postflight brought by any additional flight are anecdotal compared to the change after the first flight.

## Discussion

The aim of this study was to examine the effect of prolonged exposure to microgravity on the otolith-mediated OCR in a relatively large study sample of 27 cosmonauts. Since several of the cosmonauts engaged in multiple 6-month missions to the ISS, a total of 44 cosmonaut experiments were included in this study. The OCR at preflight showed to be consistent for test-retest and therefore the OCR can be seen as a reliable outcome variable to assess the effect of spaceflight on the otoliths.

We found a decrease in the OCR response briefly after cosmonauts had returned to Earth (*R* + 1/3). Due to the prolonged reduction of gravitational input in space, the otolith-mediated response appeared to be affected among the cosmonauts during the first week after their return (as measured at *R* + 1/3 and *R* + 4/7). Several studies suggest that the microgravity induced deconditioning of the vestibulo-spinal, vestibulo-autonomic, and otolith-mediated reflexes, are key in gaze stabilization, postural, navigational, and locomotor problems in returning cosmonauts and astronauts^3,10,21–29^. The postflight OCR decrease has been attributed to the absence of the gravity-dependent dynamic stimulation of the otoliths^4^ and has been found to persist for several days upon return to Earth^4,10,12,21,28^. The observed OCR decrease is an important finding as it is a more direct measure of otolith function compared to balance for instance. Especially for first-time flyers, a decrease of 61.38% of the otolith-mediated OCR can lead to difficulties with gaze stabilization and posture during head tilt. Cosmonauts will have difficulties with the perception of verticality, which is essential when landing, for instance, on the surface of the Moon or Mars. Besides the contribution of the otoliths to the OCR, studies suggest that the somatosensory system and other graviceptors also contribute to the OCR^17,30–33^.

A decrease in OCR response early after return is consistent with several studies^10,15,19,34,35^. However, a study performed by Clément et al. showed no significant decrease or change in OCR postflight^15^. A possible explanation for the difference between our results and theirs is the difference in vestibular stimulation. They used a static tilt with a contribution from the vertical SCCs, while we used centrifugation with only a contribution from the otoliths. But more likely is the duration of the mission, a mission of less than two weeks compared to a space mission of 6 months. Possibly, OCR decreases would only be detected during the first hours after a short-term space mission because of a faster readaptation process of the otoliths to the 1 g condition. The clear significant decrease of the OCR early postflight shown here adds to the evidence that microgravity causes an adaptation in the otolith-mediated vestibular reflexes.

There was a significant difference in OCR at the early postflight measurement (*R* + 1/3) between the different number of flights (first to fifth flight). Our findings indicate that the first-time flyers are more affected by otolith deconditioning resulting in a more significant decrease in OCR after returning to Earth. We hypothesize that the experienced flyers suffer less from this deconditioning by possibly having acquired a central adaptation from previous space missions. To our knowledge, there are no long-term studies on OCR taking flight experience into account. On the other hand, experience from previous spaceflight missions is thought to influence sensorimotor adaptations to microgravity or spaceflight^27^. Our results strongly suggest that prior spaceflight experience may facilitate vestibular adaptation to altered gravitational environments. This would be particularly advantageous for missions to the Moon and Mars, since more gravitational transitions are made during such missions compared to the current ISS missions. However, this finding raises a new question: is the mission duration of a cosmonaut’s first flight a relevant factor? It could be that a short-duration mission for the first flight is sufficient to induce the necessary adaptation instead of a 6-month mission, which may have practical benefits.

A possible countermeasure against deconditioning of otolith-mediated reflexes, such as the OCR, could be inflight centrifugation. During the 1998 Neurolab mission (STS-90), four astronauts were exposed to centripetal accelerations along the interaural axis of 0.5 and 1 g during centrifugation, both pre- and in-flight. There are two remarkable findings coming from this experiment. First, postflight tests indicated no symptoms of OI in all four astronauts. This is an unlikely occurrence, because roughly 75% of astronauts do experience symptoms of postflight OI. In addition, sympathetically mediated vasoconstriction was better maintained in comparison to astronauts who were not subjected to inflight centrifugation. Second, the degree of OCR was maintained throughout and after spaceflight, possibly due to the in-flight centrifugation, although the short mission duration (16 days) might have also contributed. This contrasts with most previous postflight OCR studies. If intermittent inflight centrifugation proves to be a countermeasure to otolith-based sympathetic, postural, and ocular deconditioning in microgravity, artificial gravity could facilitate future long-distance missions^4,11,14,36^.

The OCR of the last postflight measurement (*R* + 8/12) no longer significantly differed from the preflight measurements (BDC1). This indicates that the otoliths are recovered or readapted to the gravitational level of 1 g on Earth for all flyers within a little over a week. This finding agrees with those of two earlier studies^6,37^. The delay in the adaptation of the otolith-mediated vestibular reflex may have severe consequences for the crew after entering altered gravity levels. The current data only represent the recovery rate of 8–12 days for the vestibular response. A study performed by Demertzi et al. shows that a cosmonaut who stayed in the ISS for 6 months still showed alterations in the cortical vestibular network even after 9 days postflight^38^. In addition, Pechenkova et al. found that postflight functional connectivity changes of the anterior insula, a region known to play a role in cortical vestibular processing, were correlated with space motion sickness scores, which is also believed to be driven by the vestibular system^28^. Another study performed by Hupfeld et al. investigated how spaceflight affects the neural processing of applied vestibular stimulation using functional MRI^35^. Data were collected from 15 astronauts twice preflight and four times after their return to Earth. During preflight sessions, vestibular stimulation elicited activation of the vestibular cortex and deactivation of somatosensory and visual cortices. When the preflight measurements were compared postflight, they found reductions in the somatosensory and visual cortical deactivation, supporting sensory compensation, and reweighting with spaceflight. These observed brain changes recovered to preflight levels 3 months after spaceflight. This could suggest that the otolith-mediated vestibular reflex takes less time to recover in comparison to the vestibular processes at the cortical level.

The direction of centrifugation (CCW and CW) showed to be significant in our different models of OCR. Unfortunately, the direction of centrifugation couldn’t be randomized due to a fixed experimental protocol. Therefore this result should be interpreted with caution. The effect shown of the direction of the centrifugation could presumably be a habituation effect as CW was always performed after CCW rotation.

We found a systematically significant difference in the amount of OCR between the inner and outer eye during centrifugation, where the amount of extorsion, the eye torsion away from the nose, of the inner eye (closest to the axis of rotation) was always significantly larger than the intorsion of the outer eye, i.e. eye torsion toward the nose. After the cessation of the deceleration, the OCR was no longer present and the cyclodivergence decreased when the cosmonauts reported they felt to be back in a normal upright position. This finding agrees with several studies^39–41^, although a study performed by Diamond et al. showed the opposite of our findings^42^. During that study, the subjects were rotated with a constant velocity of 3°/s around the naso-occipital axis. The OCR was measured using photographs of the whole upper part of the face. They reported a higher degree of OCR in the ipsilateral outer eye (furthest away from the axis of rotation) in comparison to the contralateral inner eye. A possible explanation for this difference could be the result of a bias. The shifting of the baseline, which was not considered when defining the zero torsion for reference, could lead to biased results. They had a sample size of seven subjects, of which six showed a smaller degree of OCR for the inner eye, opposite to what we observe, and one showed a larger degree of OCR for the inner eye, similar to our result.

We hypothesize that the disconjugacy does not imply an asymmetry between the otoliths, because the disconjugacy is mirrored between the two centrifugation directions (CCW, CW). Possible asymmetries at the level of the otoliths have been suggested by studies on OCR during and after gravitational changes induced during parabolic flight or microgravity^11,43^. As a result of these studies, they hypothesized that the control of the otolith-mediated OCR is independent between the two eyes^29,43^. De Graaf et al. suggested that activation of the utricles generates conjugate torsional eye movements whereas activation of the sacculus was thought to be responsible for disconjugate torsional eye movements^44^.

Our explanation for this disconjugate eye movement is that the pathway of this vestibulo-ocular reflex, the OCR, runs from the superior part of the vestibular nerve to the vestibular nuclei and then crosses the midline via the medial longitudinal fasciculus to the contralateral oculomotor nerve. It is therefore a crossed vestibulo-ocular response, through which the left eye is stimulated by the right utricle and vice versa. The outer otolith experiences more centripetal acceleration because it is further away from the axis of rotation (0.0745 ± 0.0008 m)^45^. It is of the order of 10% difference. As a consequence of this crossed vestibulo-response, the inner eye will show a larger otolith-driven torsion.

From our analysis, we can conclude that the OCR can be used as a reliable and robust measurement of the effect of space on the otoliths. We found a significant difference between the pre- and postflight OCR measurements for all space flyers, with a large decrease in early postflight (*R* + 1/3). The inner eye showed a systematically greater torsion compared to the outer eye because the outer otolith, which controls the contralateral inner eye, experiences a larger centripetal acceleration. The first-time flyers had a significantly lower OCR at the first postflight measurement compared to the experienced flyers who may have acquired a central adaptation from previous space missions. This could be the reason why they are significantly less affected by microgravity from a clinical perspective. This large study sample allowed us to detect underlying physiological mechanisms of the OCR and to make more general conclusions about the physiological effect of previous flight experience to microgravity on the otolith system. Our data provide a scientific basis that could support guidelines for sending experienced space crew for challenging space missions that include multiple g-level transitions, e.g., for the upcoming Moon and eventually Mars missions, since their vestibular systems are noticeably less affected by microgravity. At 8–12 days after their return, the OCR values were no longer significantly different in comparison to the preflight recordings, indicating a full readaptation of the otoliths for all space flyers. Hence, after landing on another celestial body, operational activities should take this limiting factor into account for a little over 1 week.

## Supplementary information

Supplementary figure

Reporting Summary

## Data Availability

Data can be requested from F.L.W. or I.N. (pending scientific review and a completed material transfer agreement). Requests for the OCR data should be submitted to floris.wuyts@uantwerpen.be or NaumovIvan@gmail.com.
