# Supplementary material for: Ocular counter-roll is less affected in experienced versus novice space crew after long-duration spaceflight
Source: NPJ Microgravity. 2022 Jul 20;8:27. doi: 10.1038/s41526-022-00208-5 (PMC9300597; doi:10.1038/s41526-022-00208-5)
Supplement: Supplementary file 1 — Supplementary figure [file 41526_2022_208_MOESM1_ESM.pdf]

Manuscript: "Ocular counter-roll less affected in experienced compared to novice space crew after long-duration spaceflight"

Supplementary material

Authors: Catho Schoenmaekers[1], Chloë De Laet[1], Ludmila Kornilova[2], Dmitrii Glukhikh[2], Steven Moore[3], Hamish MacDougall[4], Ivan Naumov[2], Erik Fransen[5], Leander Wille[1], Steven Jillings[1], Floris L. Wuyts[1*]

[1]Lab for Equilibrium Investigations and Aerospace, University of Antwerp, Belgium;
[2]Institute of Biomedical Problems, Moscow, Russia;
[3]Central Queensland University, School of Engineering and Technology, Queensland, Australia;
[4]University of Sydney School of Psychology, Sydney, New South Wales, Australia; and
[5]StatUa Center for Statistics, University of Antwerp, Antwerp, Belgium.

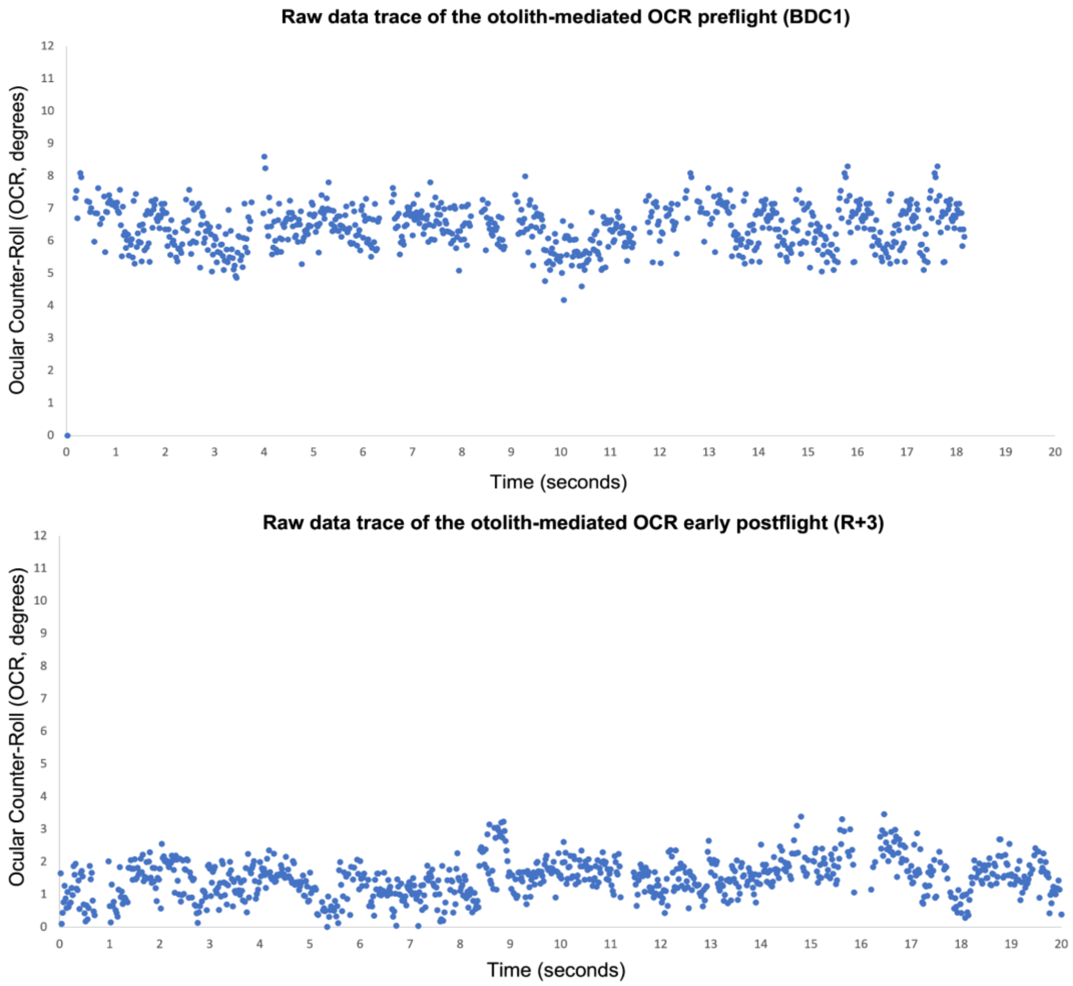

Supplementary figure Raw data trace of the otolith-mediated ocular counter-roll (OCR) pre- and postflight (BDC1 and R+3). Here we report an example of one cosmonaut's (first-time flier) OCR raw trace for one preflight (BDC1) and a three days after return (R+3) measurement.
