# Supplementary material for: Ocular counter-roll is less affected in experienced versus novice space crew after long-duration spaceflight
Source: NPJ Microgravity. 2022 Jul 20;8:27. doi: 10.1038/s41526-022-00208-5 (PMC9300597; doi:10.1038/s41526-022-00208-5)
Supplement: Supplementary file 2 — Reporting Summary [file 41526_2022_208_MOESM2_ESM.pdf]

nature portfolio

Corresponding author(s): Floris L. Wuyts

Last updated by author(s): May 24, 2022

Reporting Summary

Nature Portfolio wishes to improve the reproducibility of the work that we publish. This form provides structure for consistency and transparency in reporting. For further information on Nature Portfolio policies, see our Editorial Policies and the Editorial Policy Checklist.

Statistics

For all statistical analyses, confirm that the following items are present in the figure legend, table legend, main text, or Methods section.

n/a	Confirmed	
☐	☒	The exact sample size (*n*) for each experimental group/condition, given as a discrete number and unit of measurement
☒	☐	A statement on whether measurements were taken from distinct samples or whether the same sample was measured repeatedly
☐	☒	The statistical test(s) used AND whether they are one- or two-sided<br>*Only common tests should be described solely by name; describe more complex techniques in the Methods section.*
☐	☒	A description of all covariates tested
☐	☒	A description of any assumptions or corrections, such as tests of normality and adjustment for multiple comparisons
☐	☒	A full description of the statistical parameters including central tendency (e.g. means) or other basic estimates (e.g. regression coefficient) AND variation (e.g. standard deviation) or associated estimates of uncertainty (e.g. confidence intervals)
☐	☒	For null hypothesis testing, the test statistic (e.g. *F*, *t*, *r*) with confidence intervals, effect sizes, degrees of freedom and *P* value noted<br>*Give P values as exact values whenever suitable.*
☒	☐	For Bayesian analysis, information on the choice of priors and Markov chain Monte Carlo settings
☒	☐	For hierarchical and complex designs, identification of the appropriate level for tests and full reporting of outcomes
☒	☐	Estimates of effect sizes (e.g. Cohen's *d*, Pearson's *r*), indicating how they were calculated

Our web collection on statistics for biologists contains articles on many of the points above.

Software and code

Policy information about availability of computer code

Data collection	*Provide a description of all commercial, open source and custom code used to collect the data in this study, specifying the version used OR state that no software was used.*
Data analysis	The video files obtained during the VVIS experiment contain recordings of the eye movements and were analyzed in a visual programming language (custom made by H.M. in LabVIEW - National Instruments -11500 N Mopac 382 Expwy. Austin, TX, USA) to measure the OCR in degrees.

For manuscripts utilizing custom algorithms or software that are central to the research but not yet described in published literature, software must be made available to editors and reviewers. We strongly encourage code deposition in a community repository (e.g. GitHub). See the Nature Portfolio guidelines for submitting code & software for further information.

Data

Policy information about availability of data

All manuscripts must include a data availability statement. This statement should provide the following information, where applicable:

- Accession codes, unique identifiers, or web links for publicly available datasets
- A description of any restrictions on data availability
- For clinical datasets or third party data, please ensure that the statement adheres to our policy

Data can be requested to Floris L. Wuyts or Ivan Naumov (pending scientific review and a completed material transfer agreement). Requests for the OCR data should be submitted to: floris.wuyts@uantwerpen.be or NaumovIvan@gmail.com.

Field-specific reporting

Please select the one below that is the best fit for your research. If you are not sure, read the appropriate sections before making your selection.

☒ Life sciences ☐ Behavioural & social sciences ☐ Ecological, evolutionary & environmental sciences

For a reference copy of the document with all sections, see nature.com/documents/nr-reporting-summary-flat.pdf

Life sciences study design

All studies must disclose on these points even when the disclosure is negative.

Sample size	In total, 27 cosmonauts (13 first-time flyers, 14 experienced flyers) took part in this study, several of whom participated for multiple ISS missions (11 cosmonauts participated twice, one participated three times, and one participated four times). This resulted in a total of 44 longitudinal datasets.
Data exclusions	N/A
Replication	The second baseline measurement(BDC2) of the OCR was not different from the first one (BDC1). The OCR at preflight showed to be consistent for test-retest and therefore the OCR can be seen as a reliable outcome variable to assess the effect of spaceflight on the otoliths.
Randomization	No randomization was performed as all cosmonauts performed the same task.
Blinding	No blinding was performed as all cosmonauts performed the same task.

Reporting for specific materials, systems and methods

We require information from authors about some types of materials, experimental systems and methods used in many studies. Here, indicate whether each material, system or method listed is relevant to your study. If you are not sure if a list item applies to your research, read the appropriate section before selecting a response.

Materials & experimental systems

n/a	Involved in the study	
☒	☐	Antibodies
☒	☐	Eukaryotic cell lines
☒	☐	Palaeontology and archaeology
☒	☐	Animals and other organisms
☐	☒	Human research participants
☒	☐	Clinical data
☒	☐	Dual use research of concern

Methods

n/a	Involved in the study	
☒	☐	ChIP-seq
☒	☐	Flow cytometry
☒	☐	MRI-based neuroimaging

Human research participants

Policy information about studies involving human research participants

Population characteristics	27 Russian cosmonauts, betwen 35 and 60 years old (average = 46,32)
Recruitment	On volontary basis, the study design was presented to the cosmonaut during scientific briefing prior to the study.
Ethics oversight	The experiment protocol was designed in accordance with the ethical standards defined in the 1964 Declaration of Helsinki and was accepted by Human Research Multilateral Review Board (HRMRB), Institute for Biomedical Problems (IBMP) and European Space Agency (ESA).

Note that full information on the approval of the study protocol must also be provided in the manuscript.
